# Correlation between endogenous polyamines in human cardiac tissues and clinical parameters in patients with heart failure

**DOI:** 10.1111/jcmm.12674

**Published:** 2015-11-18

**Authors:** Clara Meana, José Manuel Rubín, Carmen Bordallo, Lorena Suárez, Javier Bordallo, Manuel Sánchez

**Affiliations:** ^1^ FarmacologíaDepartamento de MedicinaUniversidad de OviedoOviedoSpain; ^2^ Servicio de CardiologíaHospital Universitario Central de AsturiasAsturiasSpain; ^3^ Departamento de Bioquímica y Biología MolecularUniversidad de OviedoOviedoSpain; ^4^ Instituto Universitario de Oncología del Principado de AsturiasAsturiasSpain

**Keywords:** heart, atria, ornithine decarboxylase, N^1^‐acetylpolyamine oxidase, polyamines, putrescine, spermidine, spermine, cyclic AMP, heart failure

## Abstract

Polyamines contribute to several physiological and pathological processes, including cardiac hypertrophy in experimental animals. This involves an increase in ornithine decarboxylase (ODC) activity and intracellular polyamines associated with cyclic adenosine monophosphate (cAMP) increases. The aim of the study was to establish the role of these in the human heart in living patients. For this, polyamines (by high performance liquid chromatography) and the activity of ODC and N^1^‐acetylpolyamine oxidases (APAO) were determined in the right atrial appendage of 17 patients undergoing extracorporeal circulation to correlate with clinical parameters. There existed enzymatic activity associated with the homeostasis of polyamines. Left atria size was positively associated with ODC (*r* = 0.661, *P* = 0.027) and negatively with APAO‐N^1^‐acetylspermine (*r* = −0.769, *P* = 0.026), suggesting that increased levels of polyamines are associated with left atrial hemodynamic overload. Left ventricular ejection fraction (LVEF) and heart rate were positively associated with spermidine (*r* = 0.690, *P* = 0.003; *r* = 0.590, *P* = 0.021) and negatively with N^1^‐acetylspermidine (*r* = −0.554, *P* = 0.032; *r* = −0.644, *P* = 0.018). LVEF was negatively correlated with cAMP levels (*r* = −0.835, *P* = 0.001) and with cAMP/ODC (*r* = −0.794, *P* = 0.011), cAMP/spermidine (*r* = −0.813, *P* = 0.001) and cAMP/spermine (*r* = −0.747, *P* = 0.003) ratios. Abnormal LVEF patients showed decreased ODC activity and spermidine, and increased N^1^‐acetylspermidine, and cAMP. Spermine decreased in congestive heart failure patients. The trace amine isoamylamine negatively correlated with septal wall thickness (*r* = −0.634, *P* = 0.008) and was increased in cardiac heart failure. The results indicated that modifications in polyamine homeostasis might be associated with cardiac function and remodelling. Increased cAMP might have a deleterious effect on function. Further studies should confirm these findings and the involvement of polyamines in different stages of heart failure.

## Introduction

The polyamines putrescine, spermidine and spermine are aliphatic molecules ubiquitously distributed in tissues that are associated with cell replication, growth and differentiation 1, 2 and apoptosis 3. Several findings demonstrate the involvement of intracellular polyamines in cardiac physiology. They are associated with metabolic 4, 5 and functional effects in rat heart preparations in response to cardiotonic agents 6, 7. Exogenous polyamines produce different effects, depending on the compound assayed: Spermine and spermidine elicit negative inotropism 8, while putrescine produces a cardiotonic response 6, 9. These results might be explained by the fact that polyamines bind with different affinities to a variety of cytoplasmic ligands 10, 11 and many membrane proteins, including several types of ion channels 12, 13, 14 and β‐adrenoceptors, causing an increase in intracellular cAMP 6, 15, 16.

In experimental animals, polyamines have also been related to several cardiac pathological conditions and diseases, such as hypertrophy and myocardial damage in response to numerous hormonal and trophic stimuli 17, 18, 19, 20, 21. Induction of ornithine decarboxylase (ODC), the initial enzyme in the biosynthesis of polyamines, is known to occur in response to agents that induce cardiac hypertrophy 22. While overexpression of ODC in murine hearts facilitates a severe hypertrophy in case of isoproterenol stimulation of β‐adrenoceptors 23, without alteration to the ejection fraction 24. The decrease in intracellular polyamines, as a result of treatment with α‐difluoromethylornithine (DFMO), an inhibitor of ODC 25, confers protection against β‐adrenergic‐mediated cardiac hypertrophy 18, 20, 26. Therefore, a link may exist between β‐adrenoceptor stimulation and myocardial ODC activity. On the other hand, intracellular polyamines may also modulate β‐adrenoceptor‐mediated responses, as DFMO antagonizes isoproterenol‐elicited cardiotonic effect and cAMP increases 6, 16.

The finding of the involvement of polyamines in heart physiology and pathology is based on *in vivo* and *ex vivo* studies in experimental animals, and on rodent and human cultured cardiomyocytes. The lack of human studies regarding polyamine metabolism, trace amines, and cardiac function has led to the analysis of the correlation between biochemical parameters related to the synthesis and interconversion of intracellular polyamines and intracellular cAMP, determined in human heart tissue samples, with clinical and echocardiographic parameters in patients with heart failure.

## Materials and methods

### Patients and tissue samples

The study was carried out in 17 patients, 12 men (age 65–79 years old) and 5 women (age 52–82 years old). Their clinical characteristics are shown in Table 1.

**Table 1:** Percentage of occurrence of different qualitative variables in the patients included in the study

Variable	Percentage
Sex ‐ male/female	70.6/29.4
Cause of surgery Coronary/valvular/both	31.3/43.8/25
CHF	68.8
NYHA ‐ II/III	33.3/66.7
Septal wall thickness Normal/mildly/moderately abnormal	17.6/52.9/29.4
LVEF Normal/mildly/moderately abnormal	76.5/17.65/5.9
Arterial hypertension	76.5
Diabetes mellitus	37.5
Atrial fibrillation	25
Beta blockers	42.9
ACE Inhibitors	42.9
CCB	46.7
Smoking	46.7

ACE: angiotensin converting enzyme; CHF: cardiac heart failure; CCB: calcium channel blockers; NYHA: New York Heart Association; LVEF: left ventricular ejection fraction.

A piece of right atrial appendage, 714.19 ± 50.02 mg in weight, was obtained at the time of atrial cannulation (in patients undergoing extracorporeal circulation) immediately placed at 4°C in Tyrode's solution (mM composition: NaCl, 137; KCl, 2.7; CaCl_2_, 1.8; MgCl_2_, 1.05; NaH_2_PO_4_, 0.42; NaHCO_3_, 11.9 and glucose, 5.5), saturated with a 95% O_2_ and 5% CO_2_ mixture, and brought to the laboratory. The elapsed time was less than 40 min., otherwise the samples were discarded. When the sample size allowed, it was cut into several pieces (immediately frozen in liquid nitrogen, and kept at −80°C until use) to be able to perform as many of the proposed biochemical assays as possible on the same right atrial appendage from a patient. The viability of the method of preservation was studied in two patients, cutting one piece to be frozen as soon as possible, in liquid nitrogen, and another one after 40 min. in cold Tyrode's solution. There were no differences between both methods, regarding polyamines and cAMP determinations, leading to preserve the samples in Tyrode solution given its simplicity.

Human atrial samples were obtained under approval by the Regional Ethics Committee of Research, Asturias, Spain (reference 19/2003).

### Clinical parameters of the patients

The resting heart rate (beats per minute, bpm), systolic and diastolic blood pressure (mmHg), and the presence of diseases and medications of the patients were noted. Using 2D echocardiography the septal wall thickness (as reference for LV hypertrophy) and the left atrial diameter, in transversal projection, and LVEF were measured. The LVEF, septal wall thickness and left atria size were also categorized following the 2015 Guidelines of Recommendations for Cardiac Chamber Quantification by Echocardiography in Adults 27. For septal wall thickness considers normal a range 6–10 mm for male or 6–9 mm for female, mildly abnormal between 11–13 mm for male or 10–12 mm for female and moderately abnormal between 14–16 mm for male or 13–15 mm for female. Normal left atrial size was considered as between 30–40 mm for men and 27–38 mm for female, above these values the size is abnormal. For the LVEF, the cut‐off value of abnormal was of less than 52% for male or 54% for female 27.

For the clinical stages of heart failure the New York Heart Association (NYHA) criteria were followed.

### Measurement of cAMP in the right atrial appendage

To measure cAMP levels, frozen samples were homogenized using a Polytron in 1 ml of ice‐cold 10 mM Tris‐HCl pH 7.2 buffer containing 4 mM ethylenediaminetetraacetic acid (EDTA). Homogenates were boiled for 10 min. and then centrifuged at 18,000 × g for 15 min. at 4°C. Cyclic AMP in the supernatant was measured by means of a commercial radioimmunoassay kit from GE Healthcare (Little Chalfont, UK), following the manufacturer's instructions. Cyclic AMP levels were expressed as pmol/mg/protein. The protein content was determined according to the Bradford procedure.

### Ornithine decarboxylase assay in the right atrial appendage

Ornithine decarboxylase activity was determined as previously described 7, 28. ^14^CO_2_ released from L‐[1‐^14^C] ornithine, substrate of the reaction, was trapped in a filter paper and measured by liquid scintillation. The specific ODC activity was expressed as pmol of ^14^CO_2_ evolved per hour per mg of protein (pmol/hr/mg protein).

### N^1^‐acetylpolyamine oxidases assay in the right atrial appendage

For this purpose, 100–200 mg of atrial appendage were homogenized using a Polytron in 1 ml ice‐could buffer [HEPES (2‐[4‐(2‐hydroxyethyl)piperazin‐1‐yl]ethanesulfonic acid) 10 mM, sucrose 0.25 M and EDTA 1 mM, pH 7.2] four times for 10 sec. The homogenate was centrifuged for 10 min. at 550 × g, at 4°C. Then, 400 μl of the supernatant were taken and preserved at −80°C.

N^1^‐acetylpolyamine oxidases (APAO) activity was determined by a previously described method 29. Hydrogen peroxide, formed during the amine oxidase reaction, was measured spectrophotometrically by coupling 4‐aminoantipyrine with phenol in the presence of peroxidase. N^1^‐acetylspermine or N^1^‐acetylspermidine was used as substrate of the reaction. Similarly, polyamine oxidase activity was assayed using spermine as substrate instead. One unit of enzyme activity was defined as 1 nmol H_2_O_2_ formed per min. The data were expressed as mIU/mg of protein.

### Polyamine determination *via* high performance liquid chromatography in the right atrial appendage

Polyamine levels and trace amines were determined using a precolumn derivatization method as previously described 15, 30, using dansyl chloride. Quantification of polyamines was performed with 2‐hydroxydiaminopropane as an internal standard. The polyamines were expressed as nmol/mg protein.

### RT‐PCR analysis of RNA extracted from heart tissues

Total RNA was isolated from the samples of the right atrial appendage using the guanidine isothiocyanate method as previously described 31. RNA was twice reverse‐transcribed using random hexamers as primers and SuperScript^®^ reverse transcriptase (Invitrogen, Carlsbad, CA, USA) following the manufacturer's instructions. For each sample, a negative control was prepared without transcriptase.

Target cDNAs were amplified by PCR using Taq DNA polymerase (Biotools, Madrid, Spain) and pairs of specific primers for each gene product, 5′‐TACTTCCCATCGGACTC TGG‐3′ and 5′‐CATGAGTTGCC ACATTGACC‐3′ for ODC; 5′‐TT TGGAGAGCACCCCTTTTA‐3′ and 5′‐TCCAAC CCTCTTCACTGGAC‐3′ for spermidine/spermine N^1^‐acetyltransferase (SSAT) and 5′‐CAATACAGGACTC TTTCGAG‐3′ and 5′‐TTATGGTCGGAACTAACGACG‐3′ for 18S rRNA, used as an internal control for relative RT‐PCR.

Two reactions from each cDNA obtained were performed in a thermocycler (MyCycler; Bio‐Rad, Hercules, CA, USA) with an initial 4 min. denaturation step at 95°C followed by 35 cycles of 95°C for 15 sec., 55°C for 30 sec. and 72°C for 30 sec. in the case of ODC and SSAT and twenty cycles for 18S rRNA. Amplified products were separated by electrophoresis, visualized using UV after ethidium bromide staining, and photographed using a Vilber Lourmat Photodocumentation system. The size of the specific bands matched the predicted length of the amplicons. Intensity of the bands was quantified using the program PhotoCaptMw^®^ 10.1 for Windows (Vilber Lourmat, Marne‐la‐Vallée, France).

### Drugs and radiochemicals

L‐[I‐^14^C] ornithine was purchased from ICN Biomedicals (Irvine, CA, USA). Putrescine (tetramethylenediamine), spermidine (N‐[3‐aminopropyl]‐1,4‐butanediamine), spermine (*N*,*N*′‐bis[3‐aminopropyl]‐1,4‐butanediamine), N^1^‐acetylputrescine (N^1^‐Acetylputrescine hydrochloride), N^1^‐acetylspermidine (N^1^‐Acetylspermidine dihydrochloride), N^1^‐acetylspermine (N^1^‐Acetylspermine trihydrochloride), isoamylamine (isopentylamine: 1‐Amino‐3‐methylbutane) and 2‐hydroxydiaminopropane were from Sigma‐Aldrich (St Louis, MO, USA). [^3^H]AMP radioassay kit was from GE Healthcare (Little Chalfont, UK).

### Calculations and statistical analysis

The data were expressed as the means ± S.E.M. of the respective units of measure. The statistical significance between groups was calculated by means of Student's *t‐*test for unpaired data. The number of patients was at least three in each case, otherwise the results were not taken into account. The Pearson's correlation coefficient (*r*) was used to determine the linear relationship between two variables. Multiple regression analysis was performed to explore the relationship between the dependent variables (left atrial size, septal wall thickness, LVEF and heart rate) and other variables that could be used as predictors. Values of *P* ≤ 0.05 were considered as significant.

## Results

### Clinical and biochemical parameters of the patients

The clinical data of the patients are shown in the Table 1. The average values of the quantitative clinical and analytical parameters were not separated by gender (Table 2), as statistical significance were only observed for age, older female patients than male (73 ± 2.64 *versus* 65.08 ± 2.21 years, respectively, *P* = 0.046), and for putrescine and N^1^‐acetylspermidine determined in the right atrial appendage, which were lower for female. These were 0.83 ± 0.12 (*n* = 11) *versus* 0.37 ± 0.11 (*n* = 5) nmol/mg protein (*P* = 0.017) for putrescine and 34.04 ± 5.95 (*n* = 10) *versus* 11.29 ± 2.82 (*n* = 4) nmol/mg protein (*P* = 0.004) for N^1^‐acetylspermidine respectively for male *versus* female.

**Table 2:** Mean values ± S.E.M. of clinical variables of the patients and biochemical determinations in the right atrial appendage, the number of data in each case is indicated in brackets

Variable	Value (mean ± S.E.M.)
Age (17) years (male/female)	65.08 ± 2.2173 ± 2.64
Heart rate (16) beats per minute	80.86 ± 2.67
Systolic blood pressure (16) mmHg	141.33 ± 3.91
Diastolic blood pressure (16) mmHg	77.33 ± 3.3
Ventricular septal thickness (17) mm	12.06 ± 0.38
Left atria size (14) mm	42.79 ± 3.54
LVEF (17) percentage	55.49 ± 1.71
cAMP (12) pmol/mg protein	12.37 ± 1.77
ODC (13) pmol/hr/mg protein	14.67 ± 2.51
Putrescine (16) nmol/mg protein	0.64 ± 0.11
Spermidine (16) nmol/mg protein	1.08 ± 0.09
Spermine (16) nmol/mg protein	2.7 ± 0.21
N^1^‐Acetylputrescine (15) nmol/mg protein	0.38 ± 0.02
N^1^‐Acetylspermidine (14) nmol/mg protein	28.35 ± 5.47
SMO (9) mIU/mg protein	0.30 ± 0.04
APAO‐N^1^‐Acetylspermidine (8) mIU/mg protein	0.09 ± 0.01
APAO‐N^1^‐Acetylspermine (9) mIU/mg protein	0.13 ± 0.02
Isoamylamine (16) nmol/mg protein	0.48 ± 0.14

APAO: acetyl polyamine oxidase; cAMP: cyclic adenosine monophosphate; LVEF: left ventricular ejection fraction; ODC: ornithine decarboxylase; SMO: spermine oxidase; IU: international units; SEM: standard error of the mean.

### Principal component analysis and correlations of polyamines and the enzymes of synthesis and homeostasis and/or cAMP determined in the right atrial appendage

Ornithine decarboxylase activity and endogenous polyamines, determined in the right atrial appendage, were subjected to principal component analysis. The correlation matrix showed the presence of many coefficients of 0.3 and above. The Kaiser–Meyer–Olkin value was 0.64, exceeding the recommended value of 0.6 32 and Bartlett's Test of Sphericity 33 reached statistical significance (*P* = 0.027), supporting the factorability of the correlation matrix.

Principal component analysis revealed a two‐component solution explained a total of 82.35% of the variance, with component 1 contributing 57.63% and component 2 24.72% respectively. The Oblimin rotation solution revealed the presence of simple structure 34, with both components showing a number of strong loadings. There was a weak positive correlation between the two factors (*r* = 0.211) (Table 3).

**Table 3:** Pattern and Structure Matrix for principal components analysis with Oblimin Rotation of Two Factor Solution of ornithine decarboxylase (ODC) activity and endogenous polyamines determined in the right atria appendage

	Pattern matrix		Structure matrix		Communalities
	Component 1	Component 2	Component 1	Component 2	
Spermine	0.960		0.940		0.892
Spermidine	0.935		0.893		0.836
ODC	0.681	0.394	0.764	0.538	0.732
N^1^‐Acetylputrescine	0.578	0.515	0.687	0.637	0.725
Putrescine		0.984		0.958	0.933

**Figure 1:**
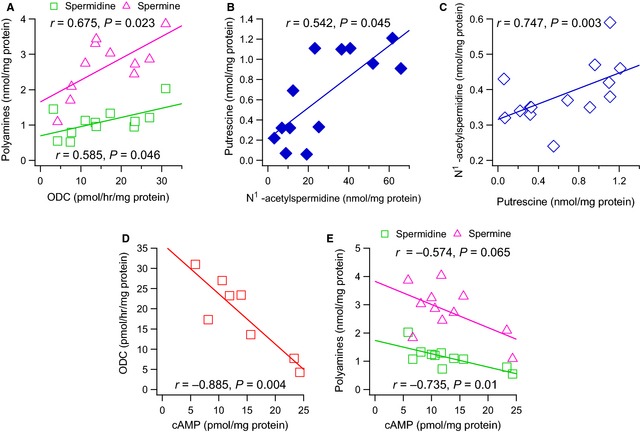
Positive correlation between ornithine decarboxylase (ODC) activity (pmol/hr/mg protein) and the polyamines (nmol/mg protein) spermidine and spermine (**A**); N^1^‐acetylspermidine and putrescine (**B**); putrescine and N^1^‐acetylputrescine (**C**) and negative correlation between cAMP (pmol/mg protein) and ODC activity (**D**) or the polyamines spermidine and spermine (**E**), determined in human right atrial appendage. The lines represent the linear regression analysis.

The linear regression analysis of the biochemical determination showed that ODC activity of the right atrial appendage positively and significantly correlated with the determinations of the polyamines spermidine (*r* = 0.585, *P* = 0.046, *n* = 12) and spermine (*r* = 0.675, *P* = 0.023, *n* = 11) (Fig. 1A), but not to putrescine (*r* = 0.405, *P* = 0.217, *n* = 11). There existed a positive correlation between the determinations of N^1^‐acetylspermidine and putrescine (*r* = 0.747, *P* = 0.003, *n* = 14) (Fig. 1B), putrescine and N^1^‐acetylputrescine (*r* = 0.542, *P* = 0.045, *n* = 14) (Fig. 1C) and between spermidine and spermine (*r* = 0.773, *P* = 0.001, *n* = 15).

Ornithine decarboxylase and APAO‐N^1^‐acetylspermidine activity tended towards a negative correlation, although it was not statistically significant (*r* = −0.617, *P* = 0.103, *n* = 8), and APAO‐N^1^‐acetylspermidine correlated negatively with spermine (*r* = −0.779, *P* = 0.023, *n* = 8).

Cyclic AMP, assayed in the right atrial appendage, negatively and significant correlated with ODC activity (*r* = −0.885, *P* = 0.004, *n* = 8) (Fig. 1D) and spermidine (*r* = −0.735, *P* = 0.010, *n* = 11), and without statistical significance with spermine (*r* = −0.574, *P* = 0.065, *n* = 11) (Fig. 1E).

### Left atria size and septal wall thickness association with polyamines and the enzymes of synthesis and homeostasis determined in the right atrial appendage

**Figure 2:**
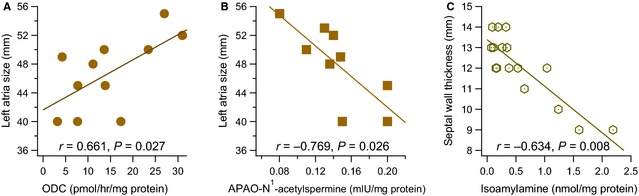
Positive correlation between left atrial size (mm) and ornithine decarboxylase (ODC) activity (pmol/hr/mg protein) (**A**) and negative between N^1^‐acetypolyamine oxidase (APAO) (mIU/mg protein), with N^1^‐acetylspermidine as substrate (**B**). Isoamylamine (nmol/mg protein) negatively correlated with septal wall thickness (**C**). The lines represent the linear regression analysis.

Left atrial size significantly correlated with ODC (*r* = 0.661, *P* = 0.027, *n* = 11) (Fig. 2A) and APAO‐N^1^‐acetylspermine (*r* = −0.769, *P* = 0.026, *n* = 8) (Fig. 2B) activity, and with the ODC/APAO‐N^1^‐acetylspermidine ratio (*r* = 0.680, *P* = 0.044, *n* = 9)

Septal wall thickness ranged from 9 to 14 mm, 3 (23.5%) patients having a normal range, 9 (47.1%) mildly abnormal and 5 (29.4%) moderately abnormal (Table 1). The patients having LV hypertrophy were significantly older, 69.14 ± 2.07 years, than those without hypertrophy, 59.33 ± 0.67 years (*P* < 0.001). They also had a significant increase in the heart rate by comparison to the patients with normal septal thickness, 82 ± 3.06 *versus* 72 ± 2.08 bpm (*P* = 0.022).

Septal wall thickness was negatively correlated with isoamylamine (*r* = −0.634, *P* = 0.008, *n* = 16) (Fig. 2C), and the one‐way anova showed significant differences (*P* < 0.001) when the variable was categorized (normal: 9.33 ± 0.33 mm, *n* = 3; mildly abnormal: 12.11 ± 0.2 mm, *n* = 9; moderately abnormal: 13.6 ± 0.24 mm, *n* = 5). Correlations with other variables were not observed.

The multivariable regression analysis did not find independent variables as predictors of left atria size or septal wall thickness.

### Patient cardiac functional parameters and its correlation with polyamines and homeostasis and/or cAMP in right atrial appendage

**Figure 3:**
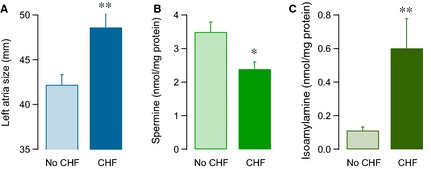
Histograms of the average value ± S.E.M. of the left atria size (mm) (**A**), spermine (nmol/mg protein) (**B**) and isoamylamine (nmol/mg protein) (**C**), regarding whether the patients present or not congestive heart failure (CHF). *P < 0.05 and **P ≤ 0.01, for unpaired data by means of the Student′s *t*‐test.

Of the 17 patients, 12 were diagnosed of congestive heart failure. These patients showed a significant increase in the left atrial size with respect to those without it (Fig. 3A). Congestive heart failure was also associated with a significant decrease in spermine levels (Fig. 3B) and an increase in isoamylamine (Fig. 3C) in the right atrial appendage.

**Figure 4:**
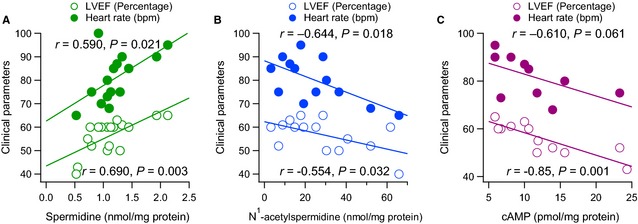
Positive correlation between spermidine (**A**) and negative for N^1^‐acetylspermidine (nmol/mg protein) (**B**) and cAMP (pmol/mg protein) (**C**) with respect to the left ventricular ejection fraction (LVEF %) and the heart rate (beats per minute: bpm) in the same patient. The lines represent the linear regression analysis.

The functional implication of polyamines on heart function was studied by the analysis of the linear correlation of the different variables with the LVEF. Spermidine (*r* = 0.690, *P* = 0.003, *n* = 16) was positively and significantly correlated with the LVEF (Fig. 4A), as well as spermine, although without statistical significance (*r* = 0.488, *P* = 0.065, *n* = 16), and negative for N^1^‐acetylspermidine (*r* = −0.554, *P* = 0.032, *n* = 15) (Fig. 4B) and APAO‐N^1^‐acetylspermidine (*r* = −0.701, *P* = 0.035, *n* = 9).

Patients that did not have preserved ejection fraction showed a significant decrease in ODC activity in atrial appendage (8.42 ± 1.95, *n* = 3 *versus* 16.55 ± 1.75, *n* = 10, *P* = 0.03) and almost a significant decrease in the ODC/cAMP ratio (0.52 ± 0.3, *n* = 3 *versus* 2.16 ± 0.41, *n* = 7, *P* = 0.069) with respect to a normal LVEF.

Spermidine (*r* = 0.590, *P* = 0.021, *n* = 15) was positively and significantly correlated with the heart rate of patients (Fig. 4A) and negatively with N^1^‐acetylspermidine (*r* = −0.644, *P* = 0.018, *n* = 13) (Fig. 4B). The LVEF and heart rate were positively and significantly correlated between each other (*r* = 0.675, *P* = 0.006, *n* = 15).

Left ventricular ejection fraction negatively and significantly correlated with cAMP (*r* = −0.850, *P* = 0.001, *n* = 13) and almost for heart rate (*r* = −0.610, *P* = 0.061, *n* = 10) (Fig. 4C). As well as LVEF with the cAMP/ODC (*r* = −0.794, *P* = 0.011, *n* = 10), cAMP/spermidine (*r* = −0.813, *P* = 0.001, *n* = 13) and cAMP/spermine (*r* = −0.747, *P* = 0.003, *n* = 13) ratios.

The multivariable regression analysis did not find independent variables as predictors of LVEF. However, spermidine, spermine and N^1^‐acetylspermine explained 75.8% of the variance in heart rate (*P* = 0.008). These independent variables also made a statistical significant unique contribution to heart rate. The equation of the model was: *Y*_*heart rate*_ = 91.03 (constant) + spermidine × 19.83+ spermine × −9.06+ N^1^‐acetylspermine × −0.32.

Patients treated with atenolol showed a decrease in the LVEF 52.67 ± 2.78% (*n* = 6) in comparison with untreated patients of 60 ± 1.32% (*n* = 8), *P* = 0.024. No significant differences were observed in the remaining variables analysed, except on age 62.5 ± 2.59 (*n* = 6) for treated patients by comparison with untreated of 70.50 ± 2.31% (*n* = 8), *P* = 0.04.

No significant differences were observed in the variables analysed regarding the NYHA stages II and III, or whether the patients had a coronary or valvular disease.

### Determination of ODC and SSAT gene expression levels in right atrial appendage

**Figure 5:**
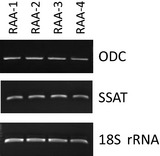
Levels of mRNA corresponding to ornithine decarboxylase (ODC) and spermidine/spermine N^1^‐acetyltransferase (SSAT) measured by RT‐PCR in human right atrial appendage; 18S rRNA was used as an internal control for relative RT‐PCR, corresponding to the patients of Table 4. RAA: atrial appendage.

RT‐PCR experiments were carried out to determine the ODC and SSAT mRNA profile. This showed that ODC and SSAT expression was similar in the 12 patients from whom tissue availability allowed the performance of RT‐PCR (Table 4; Fig. 5).

**Table 4:** Sex, age and clinical and biochemical parameters of the patients included in Figure 5

Patient	Sex	Age (years)	LAS (mm)	SWT (mm)	LVEF (%)	CHF	NYHA	ODC (pmol/hr/mg protein)
RAA‐1	Male	69	49	13	43	Yes	II	4.25
RAA‐2	Female	69	45	12	60	No	II	7.73
RAA‐3	Female	60	50	9	52	Yes	III	23.4
RAA‐4	Male	78	55	14	60	Yes	III	27

RAA: right atrial appendage; CHF: cardiac heart failure; LAS: left atria size; LVEF: left ventricular ejection fraction; LVH: left ventricular hypertrophy; NYHA: New York Heart Association; ODC: ornithine decarboxylase; SWT: septal wall thickness.

## Discussion

The results showed for the first time in humans that the polyamine pathway and trace amines, analysed in right atrial appendage, are associated with clinical and/or echocardiographic parameters of patients presenting heart failure, with regard to the effects on cardiac remodelling and function. The findings are relevant as they were obtained in heart samples taken from living patients to compare with diverse studies of cardiac hypertrophy induced in animal models, where ODC activity and polyamines play an important role in cardiac function and remodelling.

Most patients in the study had heart failure, two‐thirds were male and, as expected, females were older than male 35. The absence of values of polyamines or the activity of the enzymes related to their synthesis and interconversion in heart samples of healthy individuals (unlikely candidates for cardiac surgery with extracorporeal circulation) led us to analyse the correlation between the biochemical determinations in the right atrial appendage with respect to clinical parameters of the patients.

**Figure 6:**
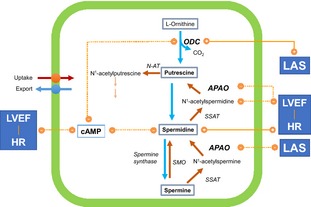
Relationship of polyamine metabolism with biochemical, functional and morphological parameters in the heart. The lines show the correlations between polyamines, enzymatic activities and cAMP with the left atrial size (LAS), the left ventricular ejection fraction (LVEF) and the heart rate (HR). (+) and solid lines positive correlation; (−) and dotted lines negative correlation. APAO: N^1^‐acetypolyamine oxidase; N‐AT: N^1^‐acetyltransferase; ODC: Ornithine decarboxylase; SMO: Spermine oxidase; SSAT: Spermidine/spermine N‐acetyltransferase.

The results of the biochemical analysis showed that the polyamine pathway is present in human heart samples of the right atrial appendage (Fig. 6).

They fitted, using factorial analysis, into two components, reflecting an interrelationship, with biological meaning, in a subset of the total variables studied. There is a first component for spermine, spermidine, ODC and N^1^‐acetylputrescine, and a second for ODC, N^1^‐acetylputrescine and putrescine. Moreover, there existed a linear correlation between polyamines and certain enzymes of the synthesis and catabolism of polyamines. Thus, this was positive and significant between ODC activity (which converts ornithine into putrescine) 36 and the levels of spermidine and spermine. Although no correlation was observed for putrescine. This could be related to the short half‐life of putrescine, converted to spermidine (by spermidine synthase) and N^1^‐acetylputrescine (*via* N‐acetyltransferase) 37, with which it is positively correlated. Putrescine may also be excreted 38, making the correlation with a specific enzyme or polyamine difficult. In the atrial appendage of these patients spermine oxidase could play a minor role in the catabolism of spermine.

In the patients studied, ODC activity and intracellular polyamines were associated with trophic and functional parameters (Fig. 6). In this sense, left atrial size was positively associated with a positive anabolic/catabolic ratio of polyamines, as shown by its positive correlation with ODC/APAO‐N^1^‐acetylspermidine activity ratio. These changes might be related to a causal effect and/or as a response to the left atrial dilation.

Left atrial size is clinically related to a sustained overload; in fact the patients with cardiac heart failure have increased left atrial size. This may be a consequence of diastolic (because of ventricular hypertrophy) and/or systolic dysfunction. With regard to diastolic dysfunction, a correlation with septal wall thickness should be expected, but this was not the case, ruling out such a possibility in these patients.

Activation of polyamine metabolism, by induction of ODC, has been proposed as an adaptive mechanism of heart through cardiac hypertrophy 39, being also associated with ventricular systolic dysfunction 24. However, our results showed that septal wall thickness was not correlated with ODC and APAO‐N^1^‐acetylspermidine activity, increased polyamine levels or changes in the expression of ODC and SSAT. This suggests that, at least, in mild to moderately abnormal septal wall thickness the association of polyamines with cardiac deleterious effect reported in experimental animals were not observed. We cannot rule out an anabolic effect of polyamines in cardiac hypertrophy in the case of severely abnormal thickness, because none of the patients fulfilled this criterion.

Left atrial overload might be a compensatory signal for increasing the synthesis/catabolism ratio of polyamines preventing systolic dysfunction in these patients. In this sense, intracellular levels of spermidine were positively correlated with LVEF and resting heart rate. Consistent with this finding, the catabolism of spermidine, taking into account the increase in N^1^‐acetylspermidine, was negatively associated with LVEF and resting heart rate. The resting heart rate could be predicted in 3/4 of patients *via* the values of spermidine, spermine and N^1^‐acetylspermidine determined in the right atrial appendage.

In patients with an abnormal LVEF ODC activity and its ratio with cAMP were significantly decreased by comparison with those having preserved function. When they had congestive heart failure a significant decrease in the intracellular levels of spermine in the right atrial appendage was observed. Overall, intracellular polyamines seem to be important for cardiac function. Similarly, the up‐regulation of polyamines synthesis contributes to preconditioning‐induced cardioprotection and attenuation of contractile dysfunction in the murine heart 40, 41.

The cellular effects of polyamines are complex. Thus, polyamines function as reactive oxygen species (ROS) scavengers and anti‐inflammatory molecules 42, 43, 44. But increased intracellular levels of polyamines are cytotoxic, being this effect prevented by the catabolic enzymes 45, 46. However, the oxidation reactions, *via* APAO, may also lead to the formation of ROS 36, which are associated with contractile dysfunction 47, 48. Arginase may also be involved in polyamine metabolism and ROS formation contributing to myocardial injury during ischemia‐reperfusion 49. Reactive oxygen species may transduce β‐adrenoceptor signalling and activate protein Kinase A (PKA), even independently of β‐adrenergic stimulation and cAMP concentration 48. Polyamines and β‐adrenoceptor system are functionally connected in experimental models 6, 16, 18, 21, 23.

In the right atrial appendage of the patients studied, cAMP was negatively associated with ODC activity, spermidine and spermine levels. Cyclic adenosine monophosphate and the cAMP/ODC, cAMP/spermidine and cAMP/spermine ratios are negatively associated with LVEF. However, cAMP levels were not related to left atrial size and ventricular septal thickness. This underlines the possibility of contractile dysfunction when cAMP increases, and that higher ODC activity and intracellular spermidine and spermine levels could improve it. An inverse association between intracellular polyamines and cAMP was also reported in primary beating heart cells of chick embryos 50. In any case, intracellular cAMP accumulation might be determinant, among other mechanisms 51, 52, 53, in adverse cardiac remodelling and deleterious function 54, 55.

It is interesting that some patients included in the study were treated with atenolol, a cardioselective β‐blocker, which should prevent the increase in intracellular cAMP, but this was not the case. This indicates that in these patients, cAMP levels might be independent of β_1_‐adrenoceptor stimulation. Heart failure, initially compensated for by sympathetic activation of β‐adrenoceptors, turned into a diminished inotropic reserve of the heart, because of an altered expression of the β_1_/β_2_‐adrenoceptors ratio, its coupling to G‐proteins and phosphodiesterases 56, 57, 58, and cAMP compartmentalization, leading to cardiotoxic signalling 59, 60, 61. Furthermore, cAMP may activate PKA eliciting contractile dysfunction and hypertrophy of the heart 47.

Besides polyamines, isoamylamine, a primary amine produced by leucine decarboxylation, was present in the right atrial appendage. This is characterized as agonists of trace amine‐associated receptors 62, but in the heart its source and function are not known. Isoamylamine negatively correlated with septal wall thickness and was increased in the patients with cardiac heart failure. According to these findings, it might be used as potential marker of cardiac diseases.

According to these results, ODC activity is associated with left atrial hemodynamic overload and polyamines with an improvement in ventricular inotropism, which might be a compensatory mechanism to preserve cardiac contractility. These associations do not necessary imply a causal relationship with cardiac pathology, or that the biochemical changes observed in the polyamine pathway in the right atrial appendage occur in the left ventricles or whether they play a role in the physiopathology of cardiac diseases. Although they can also be released from cells and produce biological effects in cardiomyocytes 63. In any case, at least the polyamines and isoamylamine may be potential markers. Further studies should establish their role in the transition from compensatory hypertrophy to dysfunction.

In addition, the pharmacological treatment of cardiac hypertrophy, as well as cancer chemotherapies, based on the inhibition of ODC and a decrease in intracellular polyamines, must be carefully considered in terms of cardiac function.

## Disclosure

The authors confirm that there are no conflicts of interest.
